# Critical role of cellular cholesterol in bovine rotavirus infection

**DOI:** 10.1186/1743-422X-11-98

**Published:** 2014-05-23

**Authors:** Jin Cui, Xinliang Fu, Jiexiong Xie, Ming Gao, Malin Hong, Yao Chen, Shuo Su, Shoujun Li

**Affiliations:** 1 College of Veterinary Medicine, South China Agricultural University, Guangzhou, Guangdong Province 510642, People’s Republic of China

**Keywords:** Bovine rotavirus, Cholesterol, Methyl-β-cyclodextrin, Infection

## Abstract

**Background:**

Bovine rotavirus (BRV) is a non-enveloped dsRNA virus that cause neonatal calf diarrhea. Lipid rafts are cholesterol-enrich membrane mircodomains that play a vital role in many cellular processes. In this study, the effect of cellular cholesterol depletion on infection of MA-104 cells with bovine rotavirus was investigated.

**Results:**

We demonstrated that cholesterol depletion of the plasma membrane by MβCD had no effect on BRV binding to cells but significantly impaired BRV entry in a dose-dependent manner and the effect was partially reversed by addition of exogenous cholesterol, suggesting the reduction of BRV infection by MβCD was specifically due to cholesterol depletion. Cholesterol depletion after virus entry did not reduce BRV replication, whereas affected virus assembly.

**Conclusions:**

Taken together, our results demonstrate that cell membrane cholesterol is essential to BRV infectivity.

## Background

Bovine Group A rotavirus (BRV), a member of the Reoviridae family, is recognized as a major agent of neonatal calf diarrhea that causes severe diarrhoea, dehydration, anorexia and death in newborn calves [1]. Rotavirus is a non-enveloped double-stranded RNA virus that consists of 6 structural (VP1-VP4, VP6 and VP7) and 5 or 6 nonstructural proteins (NSP1-6) [2,3]. Rotaviruses are classified into G and P genotypes based on the genetic variation of VP7 (Glycoprotein) and VP4 (Protease sensitive protein) outer capsid proteins, respectively [4]. To date, 27 G and 37 P types have been recognized. The G6, G8 and G10 in combination with the P (1), P (5) and P (11) genotypes are prevalent in cattle among the world [4-7]. A recent study revealed that some BRV strains could transmit directly to human infants and cause diarrhea [8]. The emergence of novel strains and the risks of interspecies transmission are of great concern.

Lipid rafts are specialized membrane microdomains that are enriched in sphingolipids, cholesterol, SRC family protein kinases, and glycosylphosphatidylinositol (GPI)-anchored proteins, cholesterol is essential in lipid raft membranes [9,10]. Lipid rafts are associated with regulation of various physiological processes, such as lipid sorting, protein trafficking [11], cell polarization and signal transduction [12]. For virus, cholesterol-enrich membrane mircodomains play an important role in multiple stages of virus life cycle, including entry, fusion, replication, assembly and budding [13-15]. Numerous studies have demonstrated that the cellular cholesterol was required for infection of many viruses, such as PRRSV [16], TGEV [17], bovine herpesvirus type 1 (BoHV-1) [18], murine leukemia virus [19] and simian virus 40 (SV40) [20], whereas influenza virus infection is independent of cell cholesterol [21]. Previous studies have found that lipid membrane microdomains are indispensable to the infection of simian, porcine and human rotaviruses [22-25]. In addition, bovine rotavirus UK strain seem to enter the cell through clathrin-mediated endocytosis [26]. However, whether cellular cholesterol impact other stages of BRV life cycle remains unclear.

In this study we explored whether cellular cholesterol depletion affects BRV infection, furthermore, the stage of BRV infection cycle which depends on cell cholesterol has also been determined. Using Methyl-β-cyclodextrin (MβCD), a pharmacological cholesterol depletion agent, we found that cellular cholesterol affected BRV entry but not virus binding, and the inhibitory effect was partially reversed by exogenous cholesterol replenishment. Intact cell membrane cholesterol was dispensable for the virus replication. However, virus assembly was in a cholesterol-dependent pathway. These results suggested that cellular cholesterol is required for BRV infection.

## Results

### Cholesterol depletion had no effect on BRV binding

**Figure 1:**
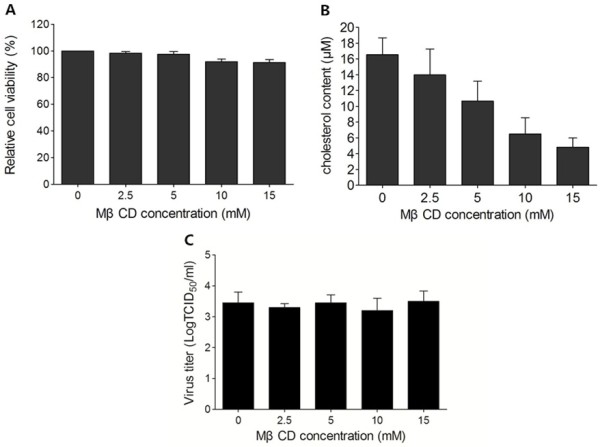
**Cellular cholesterol is not required for BRV attachment. (A)** Cell viability after treatment with various concentrations of MβCD for 30 min was determined with CCK-8 Kit. **(B)** The cellular cholesterol levels were measured by Amplex Red cholesterol assay kit. **(C)** Cholesterol depletion had no effect on BRV attachment. Cells were mock treated or treated with indicated concentrations of MβCD for 30 min, then infected with BRV at 4°C. At 1 h later, cell lysates were prepared by freeze-thaw and virus titers were measured.

In order to disrupt the lipid rafts, the cholesterol in cellular membranes was extracted by MβCD. MA-104 cells were treated with different concentrations of MβCD (0, 2.5, 5, 10 and 15 mM) for 30 min, then the cytotoxicity was measured by CCK-8 assay, the results showed that the cell survival rate (Figure 1A) did not differ significantly between treated and mock treated cells. The cholesterol contents of cellular membranes were measured by the Amplex Red cholesterol assay kit. Total cellular cholesterol levels of MβCD-treated MA-104 cells were decreased in a dose-dependent manner. As shown in Figure 1B, the amount of cholesterol following treatment with 2.5, 5, 10 or 15 mM MβCD was reduced by 15.4%, 24.9%, 60.7% or 70.7% compared with untreated cells, respectively.To test whether cholesterol depletion was able to inhibit virus attachment, virus binding assay was performed. MA-104 cells were pretreated with MβCD for 30 min, and then incubated with virus at 4°C for 1 h, virus can merely absorb to cells under this temperature. As shown in Figure 1C, no inhibitory effect was detected by virus titration assay. It indicated that cholesterol depletion had no effect on BRV attachment.

### Cholesterol depletion impaired BRV entry into MA-104 cells

**Figure 2:**
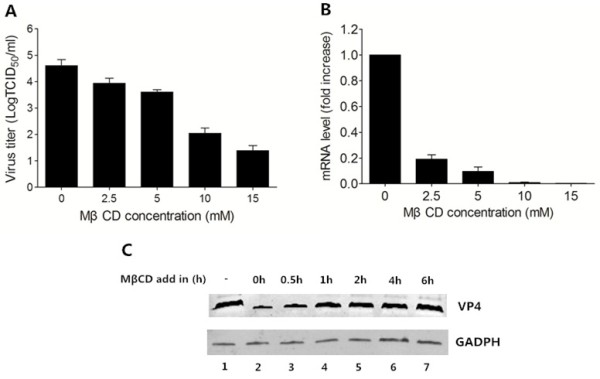
**Cholesterol depletion reduced BRV entry.** MA-104 cells were pretreated with different concentrations of MβCD followed by BRV infection for 12 h. **(A)** The virus titers were determined by TCID_50_ assay. **(B)** Viral mRNA levels were measured by Realtime PCR, results are expressed as the fold increase relative to mock-treated control (set at 1.0). All experiments were performed three times, and the error bars indicated the standard deviations of three independent experiments. **(C)** After virus attachment, the unbound BRV was removed, cells were treated with MβCD at indicated time at 37°C. At 12 hpi, the levels of VP4 in the BRV-infected cells were detected by Western blotting.

To determine the effect of cholesterol depletion on BRV entry, MA-104 cells were pretreated with various concentrations of MβCD, followed by BRV incubation. The virus production was determined at 12 hpi. The virus titers (Figure 2A) and viral mRNA levels (Figure 2B) were decreased in a dose-dependent manner. At a concentration of 15 mM, 3.22 log10 reduction in virus titer and 99.81% reduction of viral mRNA compared to mock treated cells were observed.To study at which time point cellular cholesterol became dispensable for infection, cells were exposed to MβCD during or after virus entry, as shown in Figure 2C, infection was inhibited when cells were treated during entry stage (up to 0.5 h postinternalization, Lane 2 and 3) compared to nontreated cells. However, cholesterol depletion after viral entry (1 h, 2 h, 4 h and 6 h postinternalization, lane 4 to 7) had no effect on viral protein expression. These results indicated that depletion of cellular cholesterol by MβCD impairs BRV entry into MA-104 cells.

### The inhibitive effect was partially reversed by cholesterol replenishment

**Figure 3:**
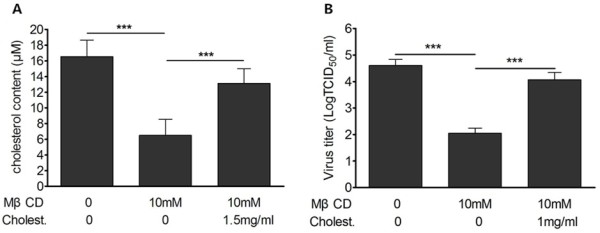
**The infection ability of BRV was partially recovered with the replenishment of exogenous cholesterol.** MA-104 cells were pretreated with 10 mM MβCD for 30 min, and then supplied with 1.5 mg/ml exogenous cholesterol for 1 h followed BRV infection. **(A)** Content of cellular cholesterol was determined with Amplex Red cholesterol assay kit. **(B)** 12 h post infection, virus titers were determined. Each experiment was performed in triplicate. “***” denotes extremely significant difference in statistics (P < 0.001).

To confirm the impairment of BRV entry by MβCD was due to depletion of cell cholesterol and whether the effect was reversible, MA-104 cells were pretreated with 10 mM MβCD for 30 min, and then suppled with 1.5 mg/ml exogenous cholesterol for 1 h, followed by BRV infection and determination of virus yield. As expected, the content of cellular cholesterol was restored to about 79.4% compared to mock treated cells (Figure 3A). The virus titers increased by 2.0 log_10_ (Figure 3B). These results demonstrated that the reduction of virus production by MβCD acts through depletion of cellular cholesterol and this effect was partially reversible.

### Effect of cellular cholesterol depletion on post-entry process

**Figure 4:**
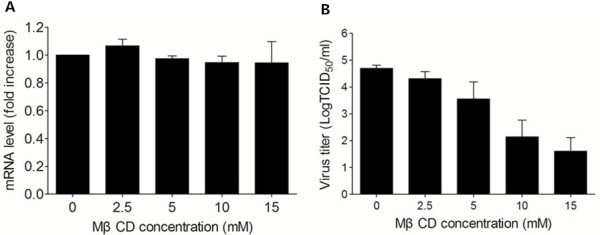
**Cholesterol depletion after virus entry did not affect BRV replication but virus assembly.** MA-104 cells were incubated with BRV for 1 h, then treated with various concentrations of MβCD, **(A)** viral mRNA levels **(B)** and virus titers were determined at 12 hpi. The results were representative of three independent experiments.

In order to analyze the effect of cellular cholesterol on post-entry stage of BRV infection, MA-104 cells were pre-incubated with BRV for 1 h at 37°C to ensure that virus has already entered into cells, and then treated with various concentrations of MβCD at 37°C for 30 min. Compared to untreated cells, viral mRNA levels (Figure 4A) and protein amounts (Figure 2C) of treated cells were not reduced, however, virus titers were significantly decreased (Figure 4B). These data shown that BRV replication on MA-104 cells is independent of cell cholesterol and cholesterol depletion impaired virus assembly.

## Discussion

Rotaviruses are the major cause of severe acute dehydrating diarrhea in humans and animals worldwide [2]. In vitro, African green monkey kidney cell (MA104) is the most commonly used cell in rotavirus propagation. Rotavirus entry is an organized multistep process, it has been demonstrated that rotaviruses enter the cell through direct penetration or by receptor-mediated endocytosis [27,28]. N-acetylneuraminic (sialic) acid residues, integrins α2β1, α4β1, αvβ3, αxβ2, heat shock cognate protein (hsc) 70 and certain gangliosides were identified as cellular molecules which were responsible for rotavirus entry [29,30]. Previous research indicated that cellular cholesterol is required for infection of some rotavirus species [22-25]. Meanwhile, cholesterol depletion impairs the entry of BRV UK strain [26]. However, BRV strains have various VP4 (P-types) and VP7 (G-types) genotypes and they were clustered into different lineages [31]. In addition, whether cholesterol affects BRV attachment or after-entry stage have not been reported. In this study, the critical role of cellular cholesterol on specific phase of BRV CH-12 strain was investigated, we found that the cellular cholesterol is sensitive to MβCD treatment. With the treatment of increasing concentrations of MβCD the cholesterol content of plasma membrane was decreased in a dose-dependent manner, and replenishing cellular membrane with exogenous cholesterol partially restored the cholesterol level. In order to determine which stage of BRV life cycle is affected by cholesterol depletion, we first examined the effect of cellular cholesterol depletion on virus binding, the results showed that BRV attachment was independent to cholesterol. However, when cells were treated with MβCD prior to virus entry, BRV infection was decreased with the reduction of cellular cholesterol level in a dose-dependent fashion. The replenishing of exogenous cholesterol enhanced the infectivity of BRV. To investigate whether cholesterol depletion affects virus entry or replication, cells were treated with MβCD after BRV entry, the result revealed that there were no significant differences of viral mRNA and protein levels between treated cells and mock cells. These results indicated that cholesterol depletion from cellular membrane did not affect the replication of BRV but virus entry. As reported, lipid raft is involved in several viruses entry into cells, such as PRRSV [16], BoHV-1 [18], human enterovirus [32], poliovirus [33] and canine distemper virus [34], cellular receptors of several viruses are localized in the lipid rafts. Although diverse rotaviruse species interact with different cell-surface molecules, some of these molecules are localized in the lipid rafts [30]. Hence, cholesterol depletion from cellular membrane influences BRV entry by disturbing lipid-raft-dependent endocytosis. Our results showed that cholesterol depletion had no effect on BRV replication, suggesting that virus replication is independent of lipid rafts, and it is in accordance with the former reports for other viruses [16,18].

Previous study demonstrated that Lovastatin-treated cells increased the levels of simian rotavirus proteins [35]. In the present work, we found that the synthesis of viral mRNA and protein were not affected when cells were treated with MβCD after virus entry, but the virus titers were obviously decreased, it appears that cholesterol-rich microdomains may contribute to assembly or budding of BRV. Lipids membrane microdomains play an important role in numerous cellular functions such as signal transduction, membrane transport and trafficking [36] and provide platforms for assembly or budding of many enveloped viruses such as influenza virus [37], HIV [38] and morbillivirus measles virus [39]. The mechanism of noneveloped viruses assembly and release is still poorly understood. The release of some noneveloped viruses from cells does not involve cell lysis. Virions transported to specific plasma membrane domains through vesicular transport, inhibition of the accumulation of virus on cell surfaces did not affect viral RNA and protein synthesis but block virus release [40,41]. Recently, compounds that could inhibit rotavirus viroplasm formation and decrease the amount of infectious progeny by disrupting lipid droplets (LDs) or fatty acid synthesis were reported [42,43]. In Caco-2 Cells, rotavirus VP4 protein has been demonstrated to directly interact with lipid microdomains. At the final stage of assembly, VP4 incorporates into preformed viral particles, lipid rafts provide a platform for the formation of infectious virions [28]. These data indicated that lipid rafts disrupted by MβCD impaired the mature of BRV particles and decreased the virus titers.

In conclusion, our results suggested that lipid rafts play a vital role in BRV infection and plasma membrane cholesterol was essential to BRV entry and assembly.

## Materials and methods

### Cells, viruses and chemicals

MA-104 African green monkey cells were maintained in DMEM containing 10% fetal bovine serum, and 1% penicillin/streptomycin. The BRV strain CH12 (kindly provided by Dr. Kun Jia, South China Agricultural University) was propagated in MA-104 cells in the presence of trypsin (final concentration was 5 mg/mL) at a multiplicity of infection (MOI) of 0.1. Methyl-β-cyclodextrin (MβCD) and cholesterol were purchased from Sigma-Aldrich (Sigma-Aldrich, St. Louis, MO, USA). The reagents were dissolved in water and diluted in serum-free DMEM.

### Depletion and replenishment of cholesterol

In order to deplete cholesterol, the cells were treated with increasing concentrations of MβCD (0, 2.5, 5, 10 and 15 mM) at 37°C for 30 min. For cholesterol replenishment, MA-104 cells were pretreated with 10 mM MβCD at 37°C for 30 min, after washing, the cells were incubated with 1.5 mg/ml exogenous cholesterol for 1 h at 37°C.

### Toxicity of MβCD and cholesterol quantification

The cell viability was measured by Cell Counting Kit (CCK-8) (Dojindo, Japan) following the manufacturer’s instructions. Briefly, MA-104 cells cultured in 96-well plates were treated without or with various concentrations of MβCD at 37°C for 30 min. Subsequently, 10 μl of the CCK-8 solution was added to each well and incubated at 37°C for 1 h, the optical density (OD) value of the wells at a wavelength of 450 nm was measured using a microplate reader (Bio-Rad, USA). Relative cell viability rate was determined for each concentration as [(OD experiment - OD blank)/(OD control - OD blank) × 100], all experiments were repeated three independent times.

To determine the cellular cholesterol level, the cholesterol-depleted or cholesterol-replenished cells were collected and diluted in 1X Amplex Red Reaction Buffer. The concentration of cholesterol was detected by the Amplex Red cholesterol Assay Kit (Life Technologies, USA) according to the manufacturer’s instructions.

### Effect of cholesterol depletion on virus binding

In order to examine if cholesterol depletion affects BRV binding to MA-104 cells, the monolayer cells seeded in 12-well plates were incubated with indicated concentrations of MβCD at 37°C for 30 min, and then infected with BRV at a multiplicity of infection (MOI) of 0.1 at 4°C for 1 h. After removing the unbound virus with PBS, cell lysates were prepared by freeze-thaw three times and virus titers were determined.

### Effect of cholesterol depletion on the BRV entry

To determine the effect of cellular cholesterol depletion on virus entry, the monolayer MA-104 cells cultured in 12-well plates were mock treated or treated with various concentrations of MβCD at 37°C for 30 min, then incubated with BRV at a MOI of 0.1 at 37°C. One hour later, cells were washed three times with PBS and supplied with serum-free medium, virus titers and viral mRNA levels were quantified at 12 hours post infection.

For cholesterol replenishment assay, the MA-104 cells were pretreated with 10 mM MβCD for 30 min, then incubated with cholesterol at a concentration of 1.5 mg/ml for 1 h. After washing, the cells were infected with BRV. At 12 hpi, virus titers were calculated.

### Add-in assay

Cells were infected with BRV at 4°C for 1 h. After binding, cells were washed with PBS to remove unbound virus, then shifted to 37°C (set as 0 h) to allow virus internalization. 30 mM MβCD was added at 0, 0.5, 1, 2, 4 and 6 h. At 12 h post infection, cells were harvested and subjected to Western blot assay.

### Effect of cholesterol depletion on post-entry stage of BRV infection

In order to analyze whether cholesterol depletion could affect virus infectivity after virus entry, MA-104 cells seeded in 12-well plates were infected with BRV for 1 h at 37°C, then mock treated or treated with various concentrations of MβCD at 37°C for 30 min. After washing, cells were kept in serum-free medium for 12 h, the virus titers and mRNA levels were determined.

### Virus titration

The monolayer MA104 cells seeded in 96-well plates were infected with serially 10-fold diluted virus. After 48 h, the level of virus infection was determined by cytopathic effect (CPE), virus titers were calculated by the Reed and Muench method and expressed as 50% tissue culture infective dose (TCID_50_) per ml.

### Realtime PCR

Total RNA was isolated using TRIzol reagent (Life Technologies, USA) and treated with RNA-free DNase (Roche) to eliminate possible DNA contamination. Reverse transcription was carried out by using Revert Aid First Strand cDNA Synthesis Kit (Thermo Scientific). The Realtime PCR was done with the SYBR Select Master Mix (Applied Biosystems, USA) according to manufacturer’s instructions. The primer set designed for BRV VP7 gene contains BRV-VP7-F (5′-TAA ATG GAT ATC AAT GGG TT-3′) and BRV-VP7-R (5′-AAC GTC AGT AAT TAC CAG C-3′), and the primer set designed for β-actin gene contains β-actin-F (5′-TCG ATC ATG AAG TGC GA CGT G-3′) and β-actin-R (5′-GTG ATC TCC TTC TGC ATC CTG TC-3′). The PCR reaction was performed with ABI7500 Fast Realtime PCR (Applied Biosystems, USA). Relative levels of viral mRNA were calculated using the 2^-ΔΔCT^ method of relative quantification with β-actin as the internal control for normalization.

### Western blot

Cells were harvested in Laemmli sample buffer and subjected to SDS-PAGE, then transferred to a nitrocellulose membrane (Millipore). The membrane was blocked with 5% nonfat dry milk in TBS and incubated with the polyclonal antibody against BRV VP4 protein, anti-rabbit IgG (H + L) (DyLight 800 Conjugate) was used as secondaryantibody (Cell Signaling). The blots were developed using LI-COR Odyssey System (LI-COR).

### Statistical analysis

The data were presented as mean ± standard deviations (SD) of three independent experiments. The differences between groups were assessed by Student’s *t* test. A P value < 0.05 was considered statistical significant. All statistical analyses were performed using GraphPad Prism.

## Competing interests

The authors declare that they have no competing interests.

## Authors’ contributions

JC, XLF and JXX carried out most of the experiments and drafted the manuscript. MG, MLH, YC and SCD participated in part of experiments. JC, SS and SJL designed the study, supervised the work and edited the final version of this manuscript. All authors have read and approved the final version of the manuscript.
